# An Algorithm for Building an Electronic Database

**Published:** 2016-01-08

**Authors:** Wess A. Cohen, Lloyd B. Gayle, Nima P. Patel

**Affiliations:** ^a^Department of Surgery, Maimonides Medical Center, Brooklyn, NY; ^b^Division of Plastic Surgery, Department of Surgery, Maimonides Medical Center, Brooklyn, NY

**Keywords:** electronic database, data collection, breast, reconstruction, database

## Abstract

**Objective:** We propose an algorithm on how to create a prospectively maintained database, which can then be used to analyze prospective data in a retrospective fashion. Our algorithm provides future researchers a road map on how to set up, maintain, and use an electronic database to improve evidence-based care and future clinical outcomes. **Methods:** The database was created using Microsoft Access and included demographic information, socioeconomic information, and intraoperative and postoperative details via standardized drop-down menus. A printed out form from the Microsoft Access template was given to each surgeon to be completed after each case and a member of the health care team then entered the case information into the database. **Results:** By utilizing straightforward, HIPAA-compliant data input fields, we permitted data collection and transcription to be easy and efficient. Collecting a wide variety of data allowed us the freedom to evolve our clinical interests, while the platform also permitted new categories to be added at will. **Conclusion:** We have proposed a reproducible method for institutions to create a database, which will then allow senior and junior surgeons to analyze their outcomes and compare them with others in an effort to improve patient care and outcomes. This is a cost-efficient way to create and maintain a database without additional software.

Developing new surgical techniques and honing established skills require not only introspection but also objective data.^1^^-^3 In accordance with the IDEAL (Idea, Development, Evaluation, Assessment and Long-term) study recommendations in 2009, institutions, attendings, and residents should track their records, cases, and outcomes to practice evidence-based learning and continually improve patient-centered care.^4^ While there are an increasing number of venues where physicians can track their outcomes, such as the American Society of Plastic Surgery's Tracking Operations and Outcomes for Plastic Surgeons (TOPS) program and the American College of Surgeons’ National Surgical Quality Improvement Program (NSQIP), building an unique database allows plastic surgeons to personalize the collected data to suite their interests and that otherwise would not be available on such platforms.^5^^,^^6^

An institutional prospectively maintained database supplies a single surgeon, institution, or surgical program a venue to track outcomes as influenced by various customizable variables. The data can assist surgeons in refining their surgical technique and improve patient outcomes and can be used to publish evidence-based results.^7^^,^^8^ In addition, a database can track and help expand patient volume and also be used to negotiate increased reimbursements as insurance companies move toward an outcomes-weighted payment system. Furthermore, an institution's database can be easily transferred to TOPS or NSQIP for nationwide data collection.

We propose an algorithm on how to create a prospectively maintained electronic database, which can then be used to analyze prospective data in a retrospective fashion and is reproducible by other institutions. The goals of a functional database are the following: (1) HIPPA compliance; (2) readily accessible by a limited number of physicians and staff from any workstation in the institution with password-protected access; and (3) easy data input and statistical output.^9^^-^12

## METHODS

**Figure 1:**
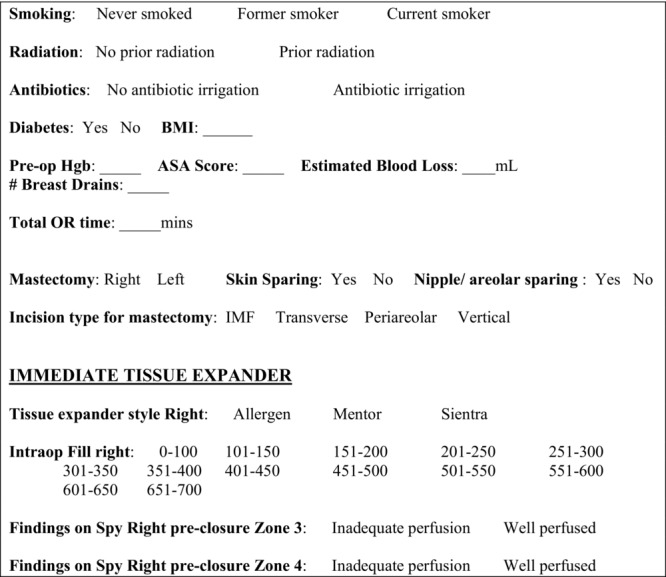
Sample OR form containing preoperative and intraoperative data. OR indicates operating room; BMI, body mass index; Hgb, hemoglobin; ASA, American Society of Anesthesiologists; and IMF, inframammary fold.

**Figure 2:**
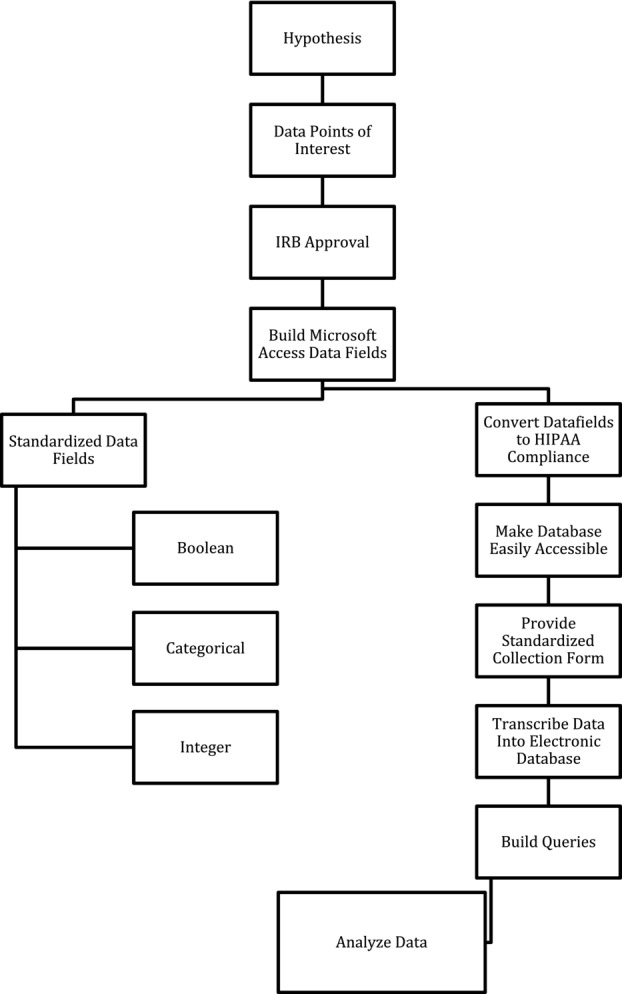
Algorithm for building an electronic database. IRB indicates institutional review board.

After institutional review board approval was obtained, we created our data fields based upon our points of interest. We included demographic and socioeconomic information, as well as intraoperative and postoperative details. Using Microsoft Access, we created drop-down menus for each data field. Drop-down menus provided standardized options that allowed easily tailored queries and were organized in a Boolean, categorical, or numerical fashion dependent on the variable. A printed form from the Microsoft Access template was given to each surgeon to be completed immediately after each case (Fig 1). The physician or the nurse practitioner then entered the case information into the database. During postoperative visits, physicians and nurse practitioners had access to the database for continued follow-up. Figure 2 outlines our algorithm.

## RESULTS

Our database was compliant with HIPPA, as all patient identifiers (ie, name, address, phone number, etc) were removed from each data set (Table 1). Also, the database was maintained on a secure password-protected server that underwent routine backups by the information technology department. By utilizing straightforward data input fields, we permitted data collection to be easy and efficient. Data were then effortlessly transcribed to Microsoft Access. Collecting a wide variety of data allowed us the freedom to evolve our clinical interests, while the platform also permitted new categories to be added at will.

## DISCUSSION

Evidence-based medicine is the most important tool physicians have to improve their practice and patient care. In 2013, the second Evidence-Based Plastic Surgery Summit recommended “that a strategic, coordinated, and sustained effort to drive an evidence-based medicine culture would accelerate adoption and advance quality of care and patient safety.”^13^ (p736) In order for physicians to truly accept evidence-based medicine, they first must understand its components. There are 5 basic elements as proposed by Swanson et al^14^:

1. Converting the need for information (eg, about prevention, diagnosis, prognosis, therapy, causation) into an answerable question.
2. Tracking down the best evidence with which to answer that question.
3. Critically appraising that evidence for its validity (closeness to the truth), impact (size of effect), and applicability (usefulness in our clinical practice).
4. Integrating the critical appraisal with our clinical expertise and with our patient's unique biology, values, and circumstances.
5. Evaluating our effectiveness and efficiency in executing steps 1 to 4 and seeking ways to improve for next time.

By maintaining a database, a surgeon can incorporate the previous elements into everyday practice: it is a tool to gather data prospectively, which can then be analyzed retrospectively. A database allows surgeons to better be able to objectively and constantly analyze their outcomes, determine the best methods and treatments for patients, and evolve their practice to ensure that they are providing the best medicine to their patients.^15^

There are limitations of a database, however. The biggest hurdle being data entry—this can be a tedious and time-consuming endeavor that is often dependent on ancillary staff.^16^ Frequently, medical record reviews are necessary to gather data, which can multiply the labor. To mitigate unnecessary work, it is important to focus tracking data of immediate interest; large data sets can become overwhelming to analyze and onerous to maintain. In addition, to query a database for statistical analysis, the data must be standardized.^16^^,^^17^ We achieved this goal by using preset drop-down menus instead of manual entry into our data fields. Although using Microsoft Access is intuitive to some, it can be overwhelming to others. As this article's purpose is not to be a primer for the program, if assistance is needed, we recommend your local information technologist or many of the comprehensive tutorials found on the Internet.

The majority of plastic surgery research falls within level IV or V evidence.^15^ The reasons for the lower grade of evidence include the need to customize reconstructions, the lack of dedicated full-time plastic surgery researchers, and the difficulty in establishing clinical trials in surgery.^1^^,^^18^ We have proposed a reproducible method for institutions to create a customizable database, which allows senior and junior surgeons to analyze their outcomes objectively and also to compare them with others in an effort to improve patient care and outcomes. Concomitantly, it provides the opportunity for residents and medical students to learn how to collect, organize, and analyze data. This is a cost-efficient way to create and maintain a database without additional software.

## CONCLUSION

Our algorithm provides future researchers a road map on how to set up, maintain, and use an electronic database to improve evidence-based care and future clinical outcomes.

## Figures and Tables

**Table 1:** HIPAA compliance

Protected health information	Shared database information
Name	Omitted
Address	Omitted
Phone number	Omitted
Medical record number	Omitted
Operating surgeon	Converted to integer
Any other unique identifying number, characteristic, or code that may be available to reidentify the individual	Omitted
